# Exploring the J-shaped relationship between HALP score and mortality in cancer patients: A NHANES 1999-2018 cohort study

**DOI:** 10.3389/fonc.2024.1388610

**Published:** 2024-09-05

**Authors:** Jiaxing Dong, Wanju Jiang, Wenjia Zhang, Taohua Guo, Yucheng Yang, Xiaohua Jiang, Liang Zheng, Tao Du

**Affiliations:** ^1^ Department of Gastrointestinal Surgery, Shanghai East Hospital, School of Medicine, Tongji University, Shanghai, China; ^2^ Department of Respiratory Medicine, Shanghai Tenth Peoples Hospital, Tongji University, Shanghai, China

**Keywords:** HALP score, cancer, non-linear association, NHANES, long-term mortality

## Abstract

**Background:**

The recent hemoglobin, albumin, lymphocyte, and platelet (HALP) scores, combined with various clinically available indicators, can comprehensively evaluate the nutritional and immune status of patients. Some observational studies have found a positive correlation between HALP score and cancer prognosis, but the clinical application of HALP score has raised concerns due to the presence of confounding factors. The aim of this study is to explore the relationship between HALP score and long-term mortality in cancer patients.

**Methods:**

We extracted 3832 cancer patients with complete baseline information from the National Health and Nutrition Examination Survey (NHANES). The COX regressions and restricted cubic spline (RCS) curves were used to explore the nonlinear relationship between HALP score and long-term mortality risk in cancer patients. Kaplan-Meier (K-M) curve was conducted to evaluate the impact of HALP score on long-term mortality risk. Additionally, subgroup analysis was conducted to verify the stability of the above results.

**Results:**

We divided participants into 4 groups based on HALP score, and the COX regression results showed that risk of long-term mortality tended to be lower in cancer patients with high HALP scores. Meanwhile, the RCS curves showed that there was a nonlinear association. The results remained stable in subgroup analyses and in breast cancer, colorectal cancer, cervix and uterus cancer, melanoma, prostate cancer and skin cancer.

**Conclusions:**

HALP score were independently associated with the risk of long-term mortality in cancer patients, and there is also a non-linear association. This will provide new perspectives on clinical and nutritional interventions for cancer patients.

## 1 Background

It is well known that systemic inflammation, anemia and nutritional deficiencies are important to the prognosis of tumors (1), and in particular inflammation was strongly associated with the tumor proliferation, metastasis and progression. Therefore, accurately assessing the immune and nutritional status of cancer patients is crucial. The hemoglobin, albumin, lymphocyte, and platelet (HALP) score is a novel immune nutritional score (2), which has been commonly used to predict prognosis in patients with malignancy (3). It integrates these factors, which can more comprehensively reflect the patient’s immune and nutritional status, thereby better guiding clinical decision-making.

In recent years, the potential value of several common inflammatory index in predicting the prognosis of cancer patients has garnered increasing attention, including the systemic immune inflammation index (SII), neutrophil-to-lymphocyte ratio (NLR), platelet-to-lymphocyte ratio (PLR), lymphocyte-to-monocyte ratio (LMR), and platelet-to-neutrophil ratio (NPR) (4–6). However, the evaluation indicators of these inflammatory indexes are relatively single and do not take into account the nutritional status of cancer patients, which restricts their clinical applicability to some extent. Furthermore, nutritional factors have been found to significantly influence cancer prognosis, as evidenced by the fibrinogen-to-albumin ratio (FAR), C-reactive protein albumin ratio (CAR), and prognostic nutritional index (PNI) (7). In comparison to these inflammatory and nutritional markers, The HALP score is considered unique and the indicators included in the HALP score are more comprehensive and can simultaneously reflect the inflammation and nutritional levels of cancer patients, thereby offering broader clinical application value. Zhao et al. investigated the significance of HALP, NLR, PLR, LMR, and the prognostic nutritional index (PNI) in predicting the prognosis and survival of breast cancer patients. The results from COX multivariate analysis indicated that only HALP emerged as an independent prognostic factor for relapse-free survival (RFS) for the early breast cancer (8). This finding suggests that HALP may offer superior predictive value for cancer prognosis compared to previous single inflammatory and nutritional indices. Similarly, in a cohort of 1,856 patients with breast cancer (BC) who underwent surgery, the HALP score has been shown to be more accurate than other commonly used inflammatory and nutritional metrics (including NLR, PLR, PNI) in predicting OS in patients with BC (9).

To date, HALP has been implemented in several clinical articles to evaluate prognosis across various sites of cancers, including gastric (10–12), colorectal (13, 14), bladder (15), pancreatic (16), prostatic (17), nephritic (18), esophageal (19), pharyngeal (20), pulmonary (21), mammary (22), and cervical cancers (23). These evidences suggest that higher HALP score tend to predict a better prognosis. However, the correlation between HALP and prognosis remains controversial due to confounding factors and tumor heterogeneity. Most observational studies have short follow-up periods and are prone to overlook potential non-linear associations. In addition, it is worth noting that most previous studies primarily focused on hospital-based, locally advanced patients, and seldom included early and terminal patients, which may introduce a certain selection bias. Furthermore, the reference value of HALP varies significantly across different studies (24). These factors collectively limit the clinical application of HALP to some extent.

The primary objective of NHANES survey is to gather comprehensive data on the health and nutrition status of both adults and children nationwide. The survey is specifically designed to encompass a representative sample of the population, making it a reliable source of information for researchers, policymakers, and health professionals. Its unique methodology combines interviews, physical examinations, and laboratory tests, offering a broad and detailed view of real-world health and nutrition. Based on the NHANES study, we investigate the association between HALP score and the long-term mortality in cancer patients, with the potential to influence clinical decision-making in cancer patients.

## 2 Methods

### 2.1 Study population and design

This retrospective cohort study obtained patient data with malignancy from the NHANES database between the years 1999 and 2018. The National Center for Health Statistics Ethical Review Board approved the project and ensured that all participants provided written informed consent.

Extensive physical examinations, including blood and urine collection, were conducted at mobile exam centers (MECs). Knowledgeable professionals from NCHS conducted interviews in participants’ homes. Initially, We initially included 5124 adult patients with cancer 5124 adult cancer patients for study. However, we excluded individuals with missing data on specific variables, such as smoking status (n = 6), education (n = 6), body mass index (BMI, n = 437), hypertension (n = 10), diabetes (n = 1), poverty income ratio (PIR, n = 401), HALP score (n = 373), and marital status (n = 19). Moreover, deaths caused by accidents were excluded (n=39). Finally, a total sample size of 3832 adults was assessed from 1999–2018.

### 2.2 Cancer status and mortality

The identification of cancer or malignancy was based on the inquiry stated in the NHANES survey questionnaires (MCQ220, MCQ230A, MCQ230B, MCQ230C): 1.”Have you ever been told by a doctor or other health professional that you had cancer or a malignancy of any kind?”; 2.”What kind of cancer was it? “. Mortality data for all participants in the study were collected by linking to the National Death Index (NDI) until December 31, 2019. The datasets contained information about mortality status and the main factors contributing to death. The ICD-10 was applied to determine the underlying causes of death. Participants who lacked sufficient identifying information or were not publicly accessible were excluded from this analysis.

### 2.3 Definition of HALP score

In the NHANES study, blood samples were collected during examinations at the MECs. The laboratory data was used to compute the HALP score, including hemoglobin (HB), lymphocyte (LYM), platelet levels, and albumin (ALB). it was calculated using the formula (25): $\text{HALP score}=\;\frac{\text{HB}\left(\text{g}/\text{l}\right)\times \text{ALB}\left(\text{g}/\text{l}\right)\times \text{LYM}\left(10^{9}/\text{l}\right)}{\text{PLT}\left(10^{9}/\text{l}\right)}$ . Based on previous studies, the HALP score was divided into four levels using quartiles (26).

### 2.4 Covariates assessment

During home interviews conducted by NHANES, a variety of information was collected through questionnaires. This information included age, gender, race, education levels, marital status, family poverty income ratio, smoking status, and disease status. The body mass index (BMI, kg/m^2^) was measured at a mobile examination center and subsequently categorized into two groups (<25kg/m^2^, ≥25 kg/m^2^).

In this subgroup analysis, we categorized race into two groups: non-hispanic white and other races (including mexican american, non-hispanic black, other hispanic and other/multiracial. Education levels were determined based on three categories: <12^th^ grade, high school grad or above, and college or above. Smoking status was divided into three categories: current smoker, former smoker, and non-smoker.

### 2.5 Statistical analysis

The collected data was statistically analyzed according to the guidelines for using NHANES data (27). The HALP score were categorized into 4 groups according to the quartiles (Q1 < 33.72; 33.72 ≤ Q2 <45.32; 45.32 ≤ Q3<61.10; Q4 ≥61.10). Since the age and HALP score in the baseline data did not conform to a normal distribution, the measurement data was described as medians and interquartile ranges, and the kruskal-wallis rank sum test was employed for comparison. For enumeration data, counts and proportions were reported, with comparisons conducting using either the chi-square test or Fisher’s exact test.

To investigate the relationship between HALP score and mortality, multivariate COX regression models were conducted to investigate the independent association after adjusting for potential confounders: Basic Model (unadjusted); Adjusted model 1 accounted for age, gender, and BMI as adjusting factors; Adjusted model 2 expanded on adjusted model 1 by including race, PIR, smoke status, hypertension, diabetes, marital status, and education level as additional adjusting factors. To check the robustness of our results, subgroup analyses stratified by some relevant effect covariates were also performed, such as age, sex, race, BMI, hypertension, enducation and cancer. In addition, ‘rms’ R package was used to build the restricted cubic spline (RCS) model to evaluate the nonlinear relationship.

Statistical significance was determined by comparing the survival probabilities using Kaplan-Meier (K-M) survival curves and log-rank test. The analysis was stratified based on clinicopathological and lifestyle factors, such as age, gender, race, BMI, smoking status, hypertension, diabetes, and education level. A two-tailed P value of less than 0.05 was used to evaluate statistical significance. The above analyses were carried out with R software (version 4.3.1: http://www.R-project.org/).

## 3 Result

### 3.1 Characteristics of the study population

**Figure 1:**
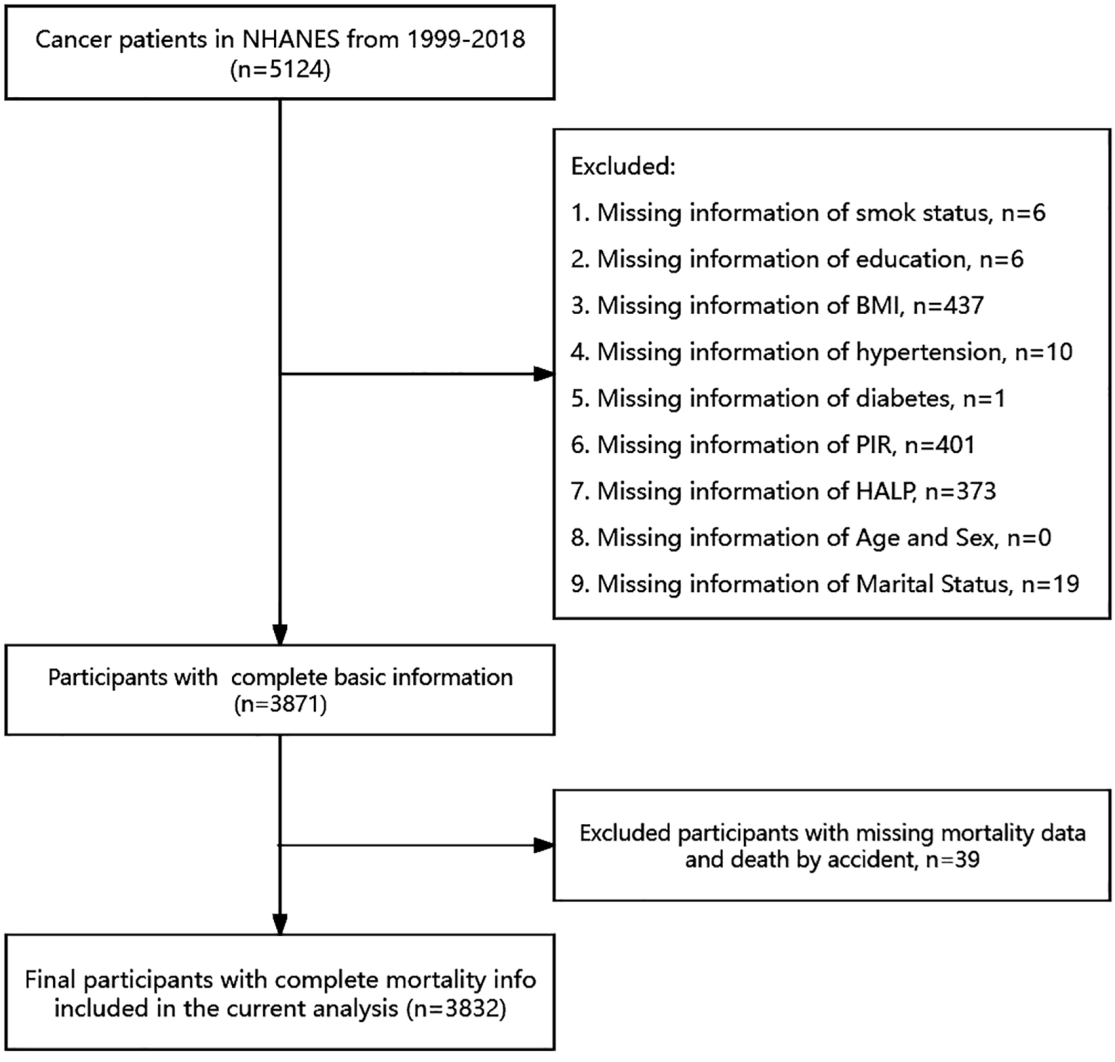
A flowchart for the inclusion and exclusion of the study population.

A total of 5124 cancer patients from the NHANES database between 1999 and 2018 were initially included. After excluding individuals with missing information on survival, smoking, education, BMI, hypertension, diabetes, PIR, HALP, marital information, and those whose deaths were triggered by accidents, a total of 3832 participants were finally included in this study (**Figure 1**). The characteristics of all participants are presented in **Table 1**. The median age of the patients was 69 years. The majority of the patients were female (53%), non-hispanic white (71%), married (58%), and had hypertension (56%). We categorized all participants into 4 groups based on quartiles of HALP score, with median HALP score of 27, 40, 52 and 74, respectively. We compared cancer patients across different HALP score levels and found significant difference in age, gender, race, smoking, BMI, marital status, and hypertension among the four HALP score groups (all *P* < 0.05).

**Table 1:** Characteristics of patients with cancer.

Characteristic	Overall (N = 3,832)	Q1 (N = 958)	Q2 (N = 958)	Q3 (N = 958)	Q4 (N = 958)	p-value
**Age (years)**	69 (57, 78)	71 (61, 80)	68 (58, 78)	68 (56, 76)	67 (54, 76)	<0.001
**HALP**	45 (34, 61)	27 (22, 31)	40 (37, 43)	52 (49, 56)	74 (67, 89)	<0.001
Gender,n(%)						<0.001
Men	1,816 (47%)	413 (43%)	430 (45%)	453 (47%)	520 (54%)	
Women	2,016 (53%)	546 (57%)	528 (55%)	505 (53%)	438 (46%)	
Race,n(%)						0.010
Non-Hispanic White	2,737 (71%)	659 (69%)	690 (72%)	683 (71%)	706 (74%)	
Non-Hispanic Black	492 (13%)	160 (17%)	121 (13%)	116 (12%)	95 (9.9%)	
Mexican American	266 (6.9%)	60 (6.3%)	67 (7.0%)	65 (6.8%)	74 (7.7%)	
Other Hispanic	182 (4.7%)	36 (3.8%)	47 (4.9%)	53 (5.5%)	46 (4.8%)	
Other/multiracial	155 (4.0%)	44 (4.6%)	33 (3.4%)	41 (4.3%)	37 (3.9%)	
Education level,n(%)						
College or above	2,106 (55%)	510 (53%)	549 (57%)	537 (56%)	510 (53%)	
GED	888 (23%)	225 (23%)	216 (23%)	224 (23%)	224 (23%)	
<= 12th	838 (22%)	224 (23%)	193 (20%)	197 (21%)	224 (23%)	
**PIR**	2.51 (1.35, 4.56)	2.46 (1.37, 4.20)	2.63 (1.37, 4.94)	2.58 (1.39, 4.62)	2.40 (1.26, 4.62)	0.13
Marital Status,n(%)						<0.001
Married	2,228 (58%)	508 (53%)	555 (58%)	580 (61%)	585 (61%)	
Widowed	667 (17%)	217 (23%)	168 (18%)	151 (16%)	132 (14%)	
Divorced/separated	601 (16%)	152 (16%)	142 (15%)	149 (16%)	158 (16%)	
Never married	336 (8.8%)	82 (8.6%)	93 (9.7%)	78 (8.1%)	83 (8.7%)	
Smoking,n(%)						<0.001
Current smoker	597 (16%)	91 (9.5%)	124 (13%)	165 (17%)	217 (23%)	
Former smoker	1,562 (41%)	446 (47%)	365 (38%)	371 (39%)	380 (40%)	
Non smoker	1,673 (44%)	422 (44%)	469 (49%)	422 (44%)	361 (38%)	
BMI,n(%)						0.038
<25.0	1,100(29%)	307 (32%)	284 (30%)	257 (27%)	253 (26%)	
>=30	1,375 (36%)	307 (32%)	346 (36%)	354 (37%)	368 (38%)	
25.00~29.99	1,357 (35%)	345 (36%)	328 (34%)	347 (36%)	337 (35%)	
Hypertension,n(%)						<0.001
No	1,702 (44%)	372 (39%)	441 (46%)	439 (46%)	450 (47%)	
Yes	2,130 (56%)	587 (61%)	517 (54%)	519 (54%)	508 (53%)	
Diabetes,n(%)						0.6
No	3,117 (81%)	780 (81%)	793 (83%)	775 (81%)	770 (80%)	
Yes	715 (19%)	179 (19%)	165 (17%)	183 (19%)	188 (20%)	

### 3.2 Univariate and multivariate Cox regression analysis between HALP score and risk of long-term mortality among cancer patients

**Figure 2:**
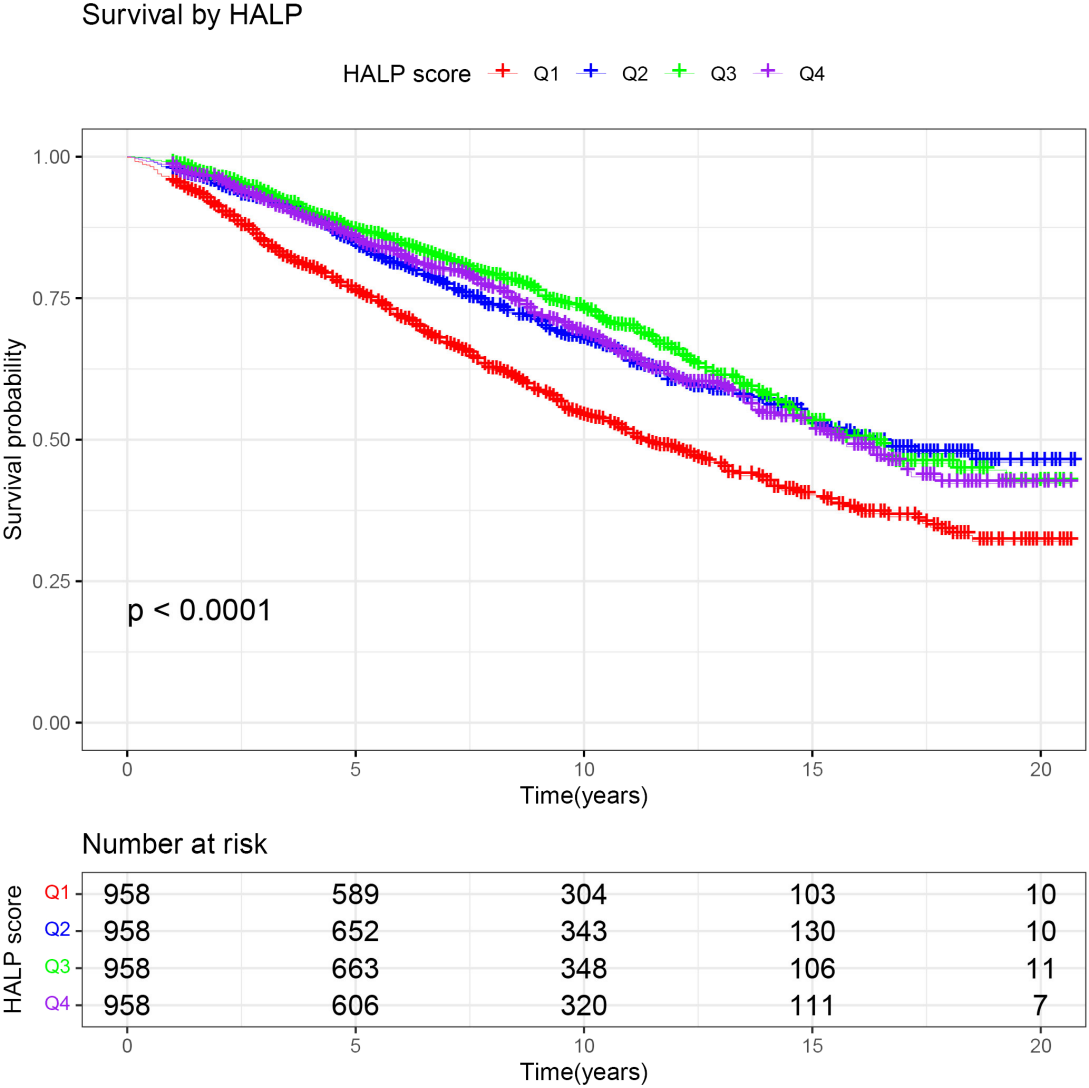
The Kaplan-Meier (K-M) curves of patients with cancer or malignant tumor.

**Figure 3:**
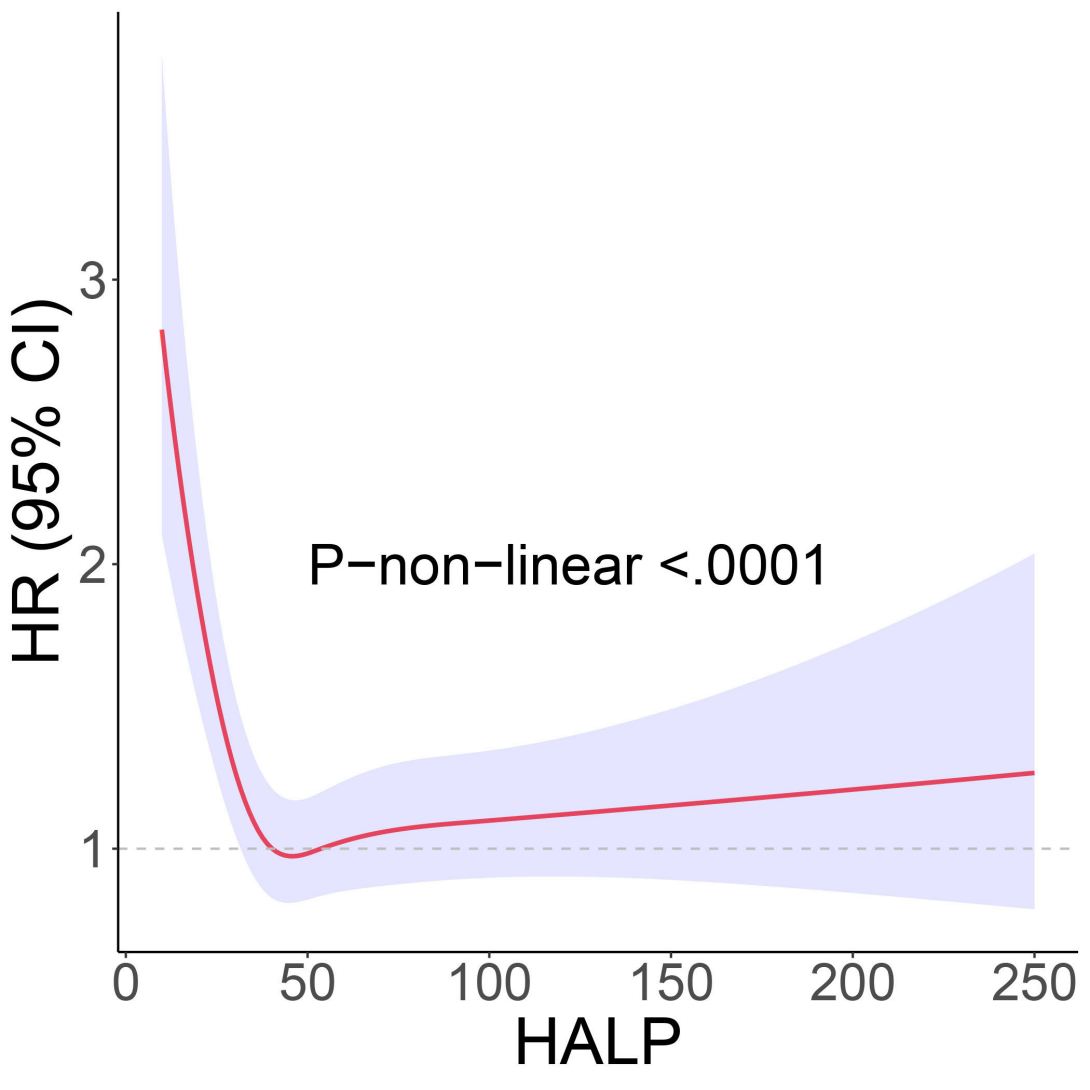
The restricted cubic spline (RCS) curve between hemoglobin, albumin, lymphocyte, and platelet (HALP) score and long-term mortality. Adjustment factors are the same as those in the adjusted model 2.

To further clarify the association between HALP and the risk of long-term mortality in cancer patients, we generated K-M curves to illustrate the survival likelihood of individuals with cancer across different HALP score (**Figure 2**), and the results indicated that higher HALP score may predict a higher survival probability in cancer patients, particularly in the Q3 group. Subsequently, we performed a univariate COX regression analysis, and the basic model showed a significant reduction in long-term mortality risk in group Q2 (HR:0.65, P<0.001), Q3 (HR:0.58, P<0.001), and Q4 (HR:0.65, P<0.001) relative to group Q1(**Table 2**). Taking into account potential confounding factors, we constructed two adjusted models, controlling for age, gender, BMI, race, PIR, smoking status, hypertension, diabetes, marital status, and education level. The results remained stable in the adjusted model. However, a higher long-term risk of death in the Q4 (Adjusted model 2: HR: 0.75) group than in the Q3 (Adjusted model 2: HR: 0.67) group was observed in both the basic and adjusted models, suggesting a potential non-linear association between HALP and the risk of death in cancer patients. Subsequently, restricted cubic spline (RCS) analysis was conducted, supporting the presence of a non-linear relationship between HALP and cancer-specific mortality (P for non-linearity<0.001) (**Figure 3**). When HALP equaled 45.75, the HR reached its lowest point. The non-linear curve showed that the HR decreased as HALP increased at lower levels. However, once reached a critical value, the HR began to rise, forming a J-shaped curve.

**Table 2:** Association between HALP score and cancer-cause mortality.

HALP Levels	Basic model			Adjusted model 1^a^			Adjusted model 2 ^b^		
	HR^1^	95% CI^1^	P-value	HR^1^	95% CI^1^	P-value	HR^1^	95% CI^1^	P-value
Q1	—	—		—	—		—	—	
Q2	0.65	0.56, 0.75	<0.001	0.74	0.64, 0.85	<0.001	0.76	0.65, 0.88	<0.001
Q3	0.58	0.50, 0.68	<0.001	0.69	0.59, 0.80	<0.001	0.67	0.57, 0.78	<0.001
Q4	0.65	0.56, 0.75	<0.001	0.79	0.68, 0.92	0.003	0.75	0.65, 0.88	<0.001

^1^HR, Hazard Ratio; CI, Confidence Interval.

a adjust for age, gender and body mass index (BMI).

b adjust for age, gender, BMI, race, poverty income ratio (PIR), Smoke status, hypertension, diabetes, marital status and education level.

### 3.3 Subgroup analysis

**Figure 4:**
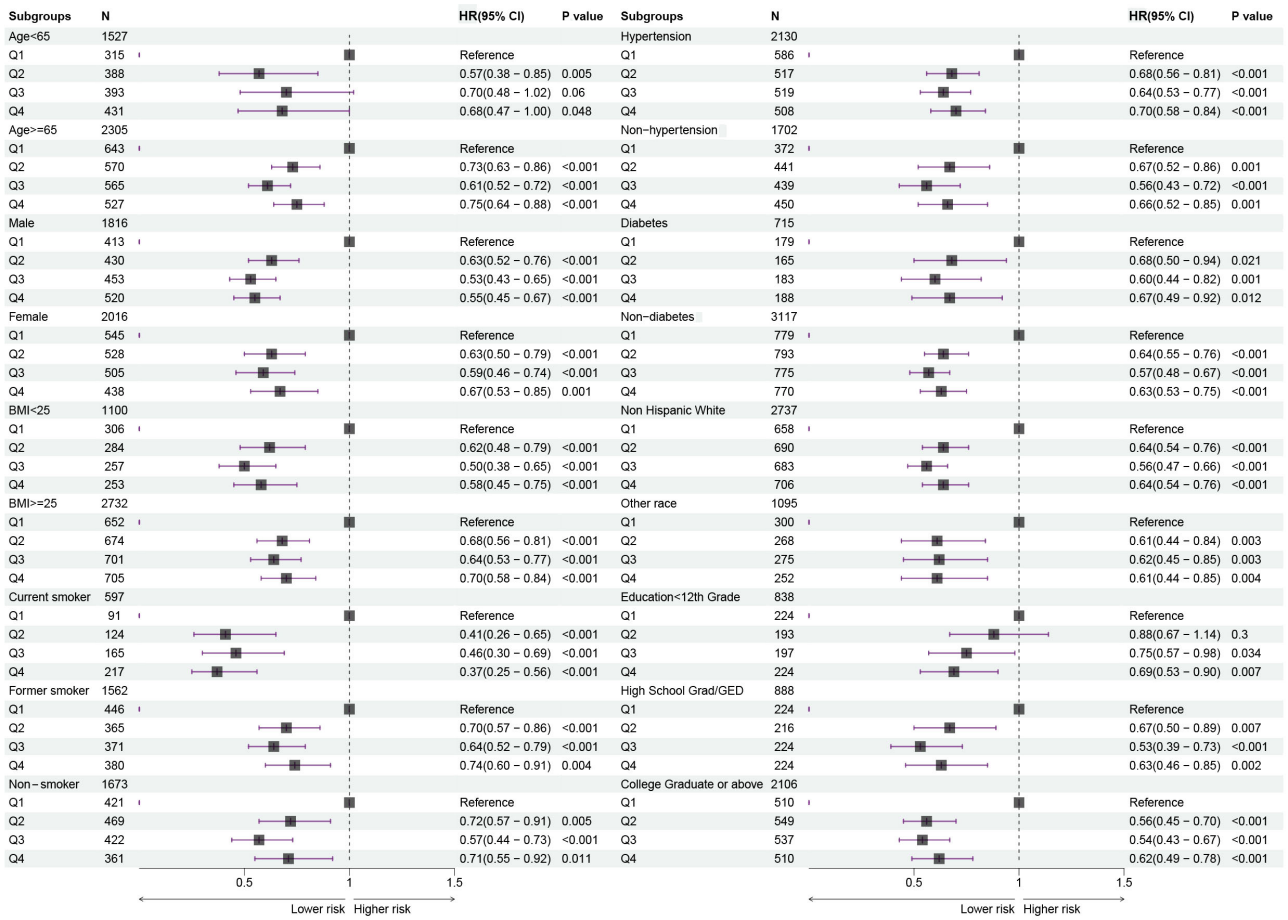
Forest plots showing the relationship between HALP score and risk of long-term mortality in subgroups.

**Figure 5:**
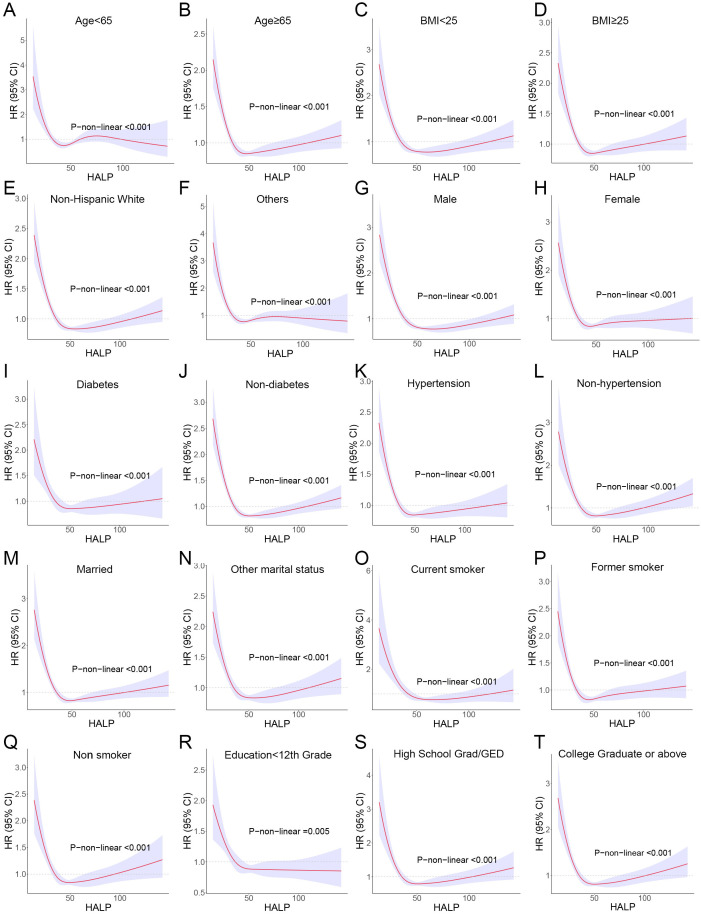
RCS curves showing the non-linear association between HALP score and risk of long-term mortality in subgroups. **(A)** Age<65; **(B)** Age<65; **(C)** body mass index (BMI) <25; **(D)** BMI≥25; **(E)** Non-Hispanic White; **(F)** Others (including Mexican American, Non-Hispanic Black, Other Hispanic and Other/multiracial. **(G)** Male; **(H)** Female; **(I)** Diabetes; **(J)** Non-diabetes; **(K)** Hypertension; **(L)** Non-hypertension; **(M)** Married; **(N)** Other marital status (including widowed, divorced/separated and never married); **(O)** Current smoker; **(P)** Former smoker; **(Q)** Non-smoker; **(R)** Education<12th; **(S)** High School Grad/GED; **(T)** College Graduate or above.

Further, we stratified for multiple factors, including age, gender, BMI, smoking, hypertension, diabetes, race, and education. The results showed that cancer patients in the high-HALP score group (Q4) had a lower risk of long-term mortality compared to the low-HALP score group (Q1) and were consistent across all subgroups (**Figure 4**). In particular, among men and current smokers, the Q4 group had the greatest reduction in long-term mortality risk compared to the Q1 group, with HR of 0.55 and 0.37. Overall, among all subgroups, the long-term mortality risk for cancer patients in the Q2, Q3, and Q4 groups was significantly lower than that in the Q1 group; however, this change was not entirely linear. Notably, we observed that the mortality risk for cancer patients in the Q4 group was higher than that in the Q3 group across most subgroups. The observed reversal phenomenon is more pronounced in specific populations, particularly among females (Q3: HR=0.59, Q4: OR=0.67), individuals with a BMI< 25 (Q3: HR=0.50, Q4: OR=0.58), non-smokers (Q3: HR=0.57, Q4: OR=0.71), those without hypertension (Q3: HR=0.56, Q4: OR=0.66), non-hispanic whites (Q3: HR=0.56, Q4: OR=0.64), and individuals who have completed high school or obtained a GED (Q3: HR=0.53, Q4: OR=0.63). In addition, the RCS curves in the subgroup analyses further confirmed that the risk of long-term mortality in cancer patients exhibits a non-linear association with HALP score (**Figure 5**).

### 3.4 Non-linear association between HALP score and risk of long-term mortality in different types of cancer

**Figure 6:**
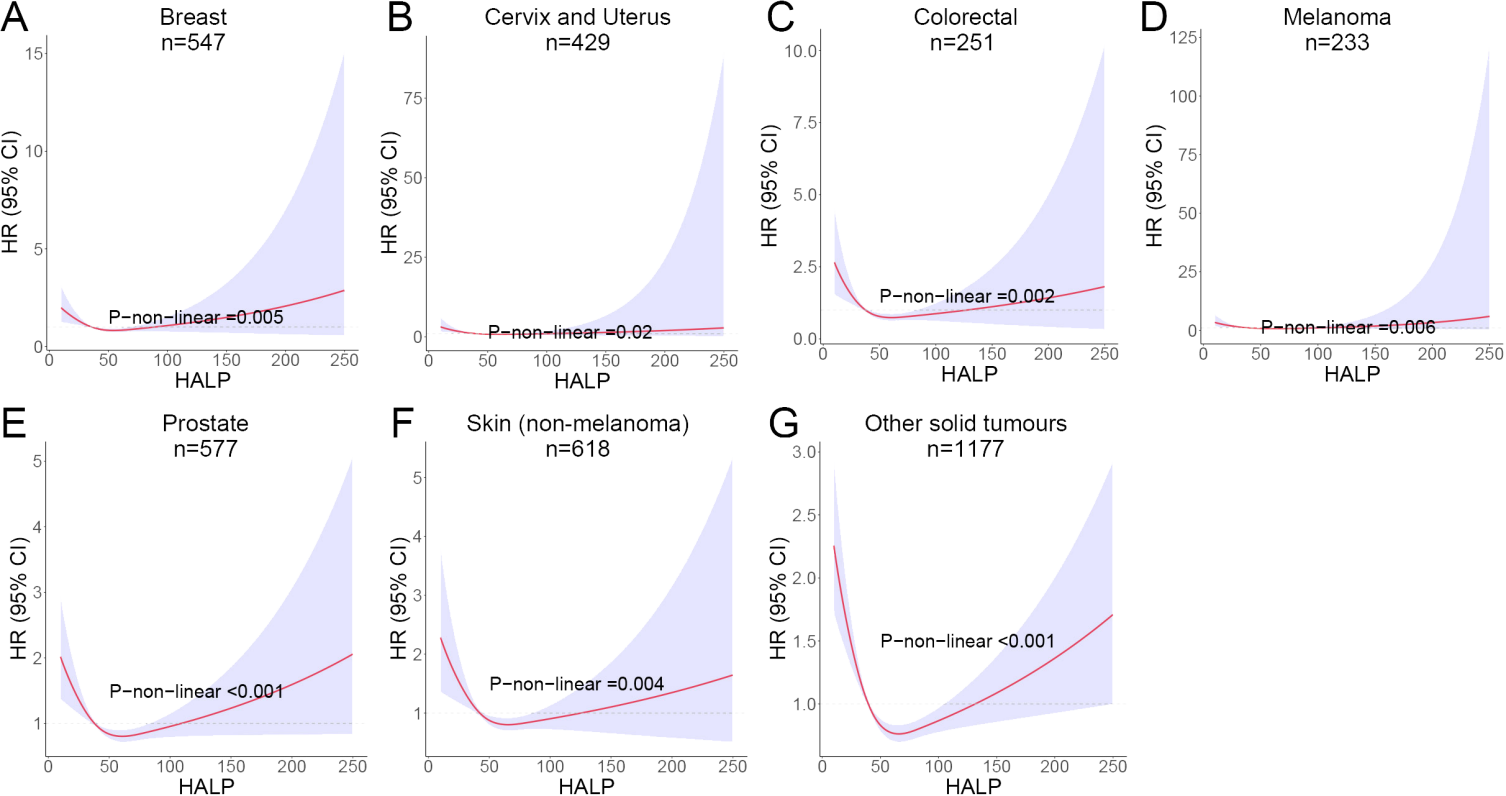
RCS curves showing the non-linear association between HALP score and risk of long-term mortality in different cancers. **(A)** Breast cancer; **(B)** Cervix and Uterus cancer; **(C)** Colorectal cancer; **(D)** Melanoma; **(E)** Prostate cancer; **(F)** Skin (non-melanoma) cancer; **(G)** Other solid tumours.

Given that the participants in this study included multiple cancer types, there may be an impact on the stability of the results. Subsequently, we performed subgroup analyses for the major cancer types with case numbers exceeding 100. The other cancers were separately divided into a group due to their small sample sizes and classification as solid tumors. Among all cancer subgroups, breast, cervix and uterine cancer patients were exclusively female, whereas prostate cancer patients were exclusively male. For the remaining cancer types, the male-to-female ratio was approximately 1:1. The baseline data for various cancer subgroups are presented in **Supplementary Table 1**. It is worth noting that a non-linear relationship between HALP score and patients’ long-term risk of mortality was still observed in breast cancer, colorectal cancer, cervix and uterus cancer, melanoma, prostate cancer and skin cancer (non-melanoma). The RCS curves also showed a J-shape pattern (**Figure 6**).

## 4 Discussion

To our knowledge, this study is the first large cohort study utilizing the NHANES database to investigate the association between blood HALP score and long-term mortality in cancer patients. In this study, we found for the first time a J-shaped relationship between blood HALP score and long-term mortality in cancer patients, with higher HALP score associated with lower long-term mortality in cancer patients within a certain range. However, once HALP values exceed the inflection point, higher HALP score instead lead to higher long-term mortality in cancer patients. This study provides new perspectives on clinical and dietary interventions to reduce long-term mortality in cancer patients.

The HALP score is a novel immunonutrition score that has gained increasing attention in recent years. It accurately predicts the prognosis of tumor patients by integrating several simple and easily accessible immune indicators (28). Among these indicators, albumin and hemoglobin levels reflect the nutritional and metabolic status of tumor patients. Lymphocytes, on the other hand, play a crucial role in the immune microenvironment, with substantial evidence confirming that lymphopenia is associated with a worse prognosis in tumor patients (29). A recent study published in *NATURE* has revealed that platelets are involved in various biological processes, including tumor angiogenesis, immune escape, chemoresistance, and promotion of distant metastasis (30). Compared to a single index, the HALP composite score provides a more comprehensive assessment of the nutritional and immune status of tumor patients. Moreover, the HALP score has been proven to be an independent prognostic factor for many cancer patients (31, 32). A gastric cancer cohort study involving 1,332 individuals found that the prognostic predictive power of the integrated HALP signature was superior to that of a nomogram constructed using TNM staging alone (33).

The HALP score is now considered as a positive indicator of prognosis and has been demonstrated in a variety of tumors, including metastatic gastric cancer (34), non-small cell lung cancer (35), endometrial cancer (36) and colorectal cancer (37). Although the value of the HALP score is well established in clinical practice, its inclusion in clinical applications remains controversial. The prior study indicated that postoperative HALP could hold significant clinical relevance. A low HALP score is associated with a poor prognosis in esophageal cancer. Feng et al. previously proposed that if a patient has a low postoperative HALP score (≤31.8), it is advisable to administer anti-inflammatory drugs or other nonsteroidal medications prior to surgery to alleviate systemic inflammation (38). This approach aims to increase hemoglobin and albumin levels to address malnutrition, thereby enhancing the prognosis for patients with esophageal cancer. For patients with LACRC undergoing radical resection, models based on the HALP score effectively identify individuals at high risk for poor survival, with a cutoff index of 26.5 (13). For patients with locally advanced cervical cancer, the authors observed that a reduced HALP (≤22.2) score independently predicts poorer oncologic outcomes (23). Additionally, the integration of the HALP index improves the precision of prognostic assessments in this patient cohort. However, due to sample size limitations and the interference of confounding factors, the HALP score varies across different tumors and populations. Furthermore, evidence regarding the impact of the HALP score on the long-term mortality risk in cancer patients remains insufficient. In this study, we conducted an analysis on a large sample population from the NHANES database. The cancer patients were categorized into four groups based on their HALP score. The results of the COX regression indicated a significant decrease in the long-term mortality risk for groups Q2, Q3, and Q4 compared to group Q1 (with the lowest HALP score). These findings align with the conclusions drawn in previous studies. Similarly, a meta-analysis incorporating 28 studies across various cancer types observed that low HALP score were associated with worse overall survival (OS) and progression-free survival (PFS) in cancer patients (39). However, unlike previous studies, the COX regression and KM curves analysis revealed that the Q3 group had a significantly lower risk of long-term mortality compared to the Q2 and Q4 groups. Subsequently, we discovered a previously unidentified nonlinear association between HALP score and long-term mortality in cancer patients. When the HALP score exceeded a certain threshold, the risk of long-term mortality in cancer patients increased.

When evaluating the HALP score, it is important to take into account the patient’s age and gender. Studies have demonstrated that as age increases, HALP tends to decrease (40). Additionally, it is worth noting that women generally have lower baseline hemoglobin levels compared to men, which can result in lower HALP score for women (41). Subgroup analyses were conducted to examine the impact of gender and age on the association between HALP score and long-term mortality in cancer patients. The results were not confounded by sex and age, with a significant non-linear relationship between HALP score and long-term mortality risk still observed. In addition, we analyzed subgroups for drink, smoking status, educational attainment and race, and the results remained consistent.

Given that the study cohort included a diverse range of solid tumors, and the heterogeneity between tumors may have had an impact on the stability of the results. Therefore, we also performed subgroup analyses for the six main solid tumors. A non-linear association between HALP and long-term mortality risk was also found in breast cancer, colorectal cancer, cervix and uterus cancer, melanoma, prostate cancer and skin cancer.

The J-shaped relationship observed between HALP scores and long-term mortality suggests a complex interplay between nutritional status, immune function, and cancer progression. Lower HALP scores reflect poor nutritional status and compromised immunity, which can lead to decreased resilience against tumor progression. While a high HALP score is generally considered favorable, an excessively elevated HALP score may indicate underlying pathological conditions and imbalances in immunonutrition. Evidence from the Danish General Suburban Population Study (GESUS) shows a non-linear relationship between platelet count and mortality (42). Within normal limits, elevated platelet counts are linked to a higher risk of cardiovascular disease, whereas reduced platelet counts are associated with an increased risk of cancer. Interestingly, while numerous studies have linked elevated platelet counts to poorer prognoses in cancer patients, there is also evidence suggesting that low platelet counts may increase the risk of cancer (43). Furthermore, a study published in the authoritative journal *BLOOD* observed a similar phenomenon, revealing that abnormal platelet counts exhibited a U-shaped association with all-cause mortality (44). This finding indicates that platelets play a complex role in cancer progression. The identification of this non-linear relationship underscores the importance of maintaining a nutritional immune balance in clinical interventions. For patients with low HALP scores, strategies aimed at enhancing nutritional intake and modulating immune function may prove beneficial, potentially involving dietary modifications, supplements, and immunotherapy. Conversely, for patients with high HALP scores, clinicians should exercise caution and refrain from assuming that a higher HALP score will necessarily correlate with a better prognosis. Maintaining HALP within a moderate range may further enhance patient outcomes.

This study is the first to investigate the association between HALP score and long-term mortality risk in cancer patients using the data from the NHANES database. The study has several strengths. Firstly, it is a cohort study that includes a large population of patients from the NHANES systematic sample. Secondly, the study utilizes three COX models and adjusts for multiple confounders. Thirdly, the results remain consistent even after conducting subgroup analyses considering various variables such as age, sex, education, drinking, and smoking status.

However, there are some limitations to this study. Firstly, we were unable to obtain specific clinical information about cancer patients, such as TNM stage and tumor size. Secondly, our study is based on the population in the USA, which somewhat limits the generalization of this study to other populations. Thirdly, despite stratifying the tumors and controlling for confounders, potential interference from other confounding factors may still exist. Furthermore, Some unmeasured confounding factors, such as detailed treatment plans and genetic variations, may potentially affect the stability of results, which were not captured in the NHANES dataset. Further clinical cohort studies are necessary to validate these findings and to explore the impact of these potential factors.

## 5 Conclusions

Our study demonstrated that HALP score are independently associated with the risk of long-term mortality in cancer patients. Interestingly, we also observed a non-linear association between HALP and the risk of cancer mortality after stratifying by multiple factors. These findings suggest that HALP score can offer valuable insights for clinical and nutritional interventions in oncology patients.

## Data Availability

The original contributions presented in the study are included in the article/**Supplementary Material**. Further inquiries can be directed to the corresponding authors.
