# Supplementary material for: Exploring the J-shaped relationship between HALP score and mortality in cancer patients: A NHANES 1999-2018 cohort study
Source: Front Oncol. 2024 Sep 5;14:1388610. doi: 10.3389/fonc.2024.1388610 (PMC11410770; doi:10.3389/fonc.2024.1388610)
Supplement: Supplementary file 1 [file Table1.docx]

**Supplementary Table.1 Characteristics of patients across different cancer**

| **Characteristic** | **Breast (**N = 547) | **CRC (**N = 251) | **Cervix-terus (**N = 429) | **Prostate (**N = 577) | **Skin (**N = 618) | **melanoma** (N = 233) | **Other cancer (**N = 1177) | **p-value** |
| --- | --- | --- | --- | --- | --- | --- | --- | --- |
| **Age (years)** | 69 (60, 77) | 73 (65, 80) | 52 (39, 63) | 74 (67, 80) | 70 (59, 79) | 71 (58, 78) | 67 (54, 77) | <0.001 |
| **Gender,n(%)** |  |  |  |  |  |  |  | <0.001 |
| Men | 0 (0%) | 132 (53%) | 0 (0%) | 577 (100%) | 346 (56%) | 123 (53%) | 638 (54%) |  |
| Women | 547 (100%) | 119 (47%) | 429 (100%) | 0 (0%) | 272 (44%) | 110 (47%) | 539 (46%) |  |
| **Race,n(%)** |  |  |  |  |  |  |  | <0.001 |
| Non-Hispanic White | 349 (64%) | 169 (67%) | 264 (62%) | 320 (55%) | 579 (94%) | 213 (91%) | 843 (72%) |  |
| Non-Hispanic Black | 81 (15%) | 42 (17%) | 49 (11%) | 176 (31%) | 9 (1.5%) | 5 (2.1%) | 130 (11%) |  |
| Mexican American | 53 (9.7%) | 19 (7.6%) | 62 (14%) | 29 (5.0%) | 12 (1.9%) | 7 (3.0%) | 84 (7.1%) |  |
| Other Hispanic | 31 (5.7%) | 13 (5.2%) | 31 (7.2%) | 27 (4.7%) | 10 (1.6%) | 4 (1.7%) | 66 (5.6%) |  |
| Other/multiracial | 33 (6.0%) | 8 (3.2%) | 23 (5.4%) | 25 (4.3%) | 8 (1.3%) | 4 (1.7%) | 54 (4.6%) |  |
| **Education level,n(%)** |  |  |  |  |  |  |  | <0.001 |
| College or above | 305 (56%) | 110 (44%) | 188 (44%) | 308 (53%) | 425 (69%) | 156 (67%) | 614 (52%) |  |
| GED | 123 (22%) | 60 (24%) | 112 (26%) | 134 (23%) | 120 (19%) | 47 (20%) | 292 (25%) |  |
| <= 12th | 119 (22%) | 81 (32%) | 129 (30%) | 135 (23%) | 73 (12%) | 30 (13%) | 271 (23%) |  |
| **PIR** | 2.42 (1.31, 4.45) | 2.19 (1.18, 3.79) | 1.68 (0.86, 3.34) | 2.64 (1.57, 4.44) | 3.51 (1.87, 5.00) | 2.95 (1.51, 5.00) | 2.33 (1.31, 4.48) | <0.001 |
| **Marital Status,n(%)** |  |  |  |  |  |  |  | <0.001 |
| Married | 266 (49%) | 133 (53%) | 189 (44%) | 401 (69%) | 400 (65%) | 153 (66%) | 686 (58%) |  |
| Widowed | 149 (27%) | 64 (25%) | 62 (14%) | 68 (12%) | 103 (17%) | 36 (15%) | 185 (16%) |  |
| Divorced/separated | 93 (17%) | 42 (17%) | 100 (23%) | 77 (13%) | 76 (12%) | 28 (12%) | 185 (16%) |  |
| Never married | 39 (7.1%) | 12 (4.8%) | 78 (18%) | 31 (5.4%) | 39 (6.3%) | 16 (6.9%) | 121 (10%) |  |
| **Smoking,n(%)** |  |  |  |  |  |  |  | <0.001 |
| Current smoker | 59 (11%) | 28 (11%) | 152 (35%) | 58 (10%) | 68 (11%) | 39 (17%) | 193 (16%) |  |
| Former smoker | 170 (31%) | 117 (47%) | 108 (25%) | 293 (51%) | 271 (44%) | 95 (41%) | 508 (43%) |  |
| Non smoker | 318 (58%) | 106 (42%) | 169 (39%) | 226 (39%) | 279 (45%) | 99 (42%) | 476 (40%) |  |
| **BMI,n(%)** |  |  |  |  |  |  |  | <0.001 |
| <25.0 | 172 (31%) | 55 (22%) | 116 (27%) | 156 (27%) | 186 (30%) | 76 (33%) | 339 (29%) |  |
| 25.00~29.99 | 165 (30%) | 99 (39%) | 111 (26%) | 227 (39%) | 239 (39%) | 79 (34%) | 437 (37%) |  |
| >=30 | 210 (38%) | 97 (39%) | 202 (47%) | 194 (34%) | 193 (31%) | 78 (33%) | 401 (34%) |  |
| **Hypertension,n(%)** |  |  |  |  |  |  |  | <0.001 |
| Yes | 309 (56%) | 168 (67%) | 194 (45%) | 377 (65%) | 324 (52%) | 120 (52%) | 638 (54%) |  |
| No | 238 (44%) | 83 (33%) | 235 (55%) | 200 (35%) | 294 (48%) | 113 (48%) | 539 (46%) |  |
| **Diabetes,n(%)** |  |  |  |  |  |  |  | <0.001 |
| No | 442 (81%) | 191 (76%) | 366 (85%) | 446 (77%) | 528 (85%) | 194 (83%) | 950 (81%) |  |
| Yes | 105 (19%) | 60 (24%) | 63 (15%) | 131 (23%) | 90 (15%) | 39 (17%) | 227 (19%) |  |
| **HALP** | 42 (31, 55) | 44 (31, 56) | 46 (35, 60) | 45 (32, 60) | 48 (37, 65) | 49 (37, 65) | 46 (34, 63) | <0.001 |
